# Immune Related Adverse Events of the Thyroid – A Narrative Review

**DOI:** 10.3389/fendo.2022.886930

**Published:** 2022-05-26

**Authors:** Christopher A. Muir, Venessa H. M. Tsang, Alexander M. Menzies, Roderick J. Clifton-Bligh

**Affiliations:** ^1^Faculty of Medicine and Health, The University of Sydney, Sydney, NSW, Australia; ^2^Cancer Genetics, Kolling Institute of Medical Research, Sydney, NSW, Australia; ^3^Department of Endocrinology, Royal North Shore Hospital, Sydney, NSW, Australia; ^4^Melanoma Institute Australia, The University of Sydney, Sydney, NSW, Australia; ^5^Department of Medical Oncology, Royal North Shore Hospital, Sydney, NSW, Australia; ^6^Department of Medical Oncology, Mater Hospital, Sydney, NSW, Australia

**Keywords:** immune related adverse events, checkpoint inhibitor, thyrotoxicosis, hypothyroidism, thyroid

## Abstract

Immune checkpoints are small molecules present on the cell surface of T-lymphocytes. They maintain self-tolerance and regulate the amplitude and duration of T-cell responses. Antagonism of immune checkpoints with monoclonal antibodies (immune checkpoint inhibitors) is a rapidly evolving field of anti-cancer immunotherapy and has become standard of care in management of many cancer subtypes. Immune checkpoint inhibition is an effective cancer treatment but can precipitate immune related adverse events (irAEs). Thyroid dysfunction is the most common endocrine irAE and can occur in up to 40% of treated patients. Both thyrotoxicosis and hypothyroidism occur. The clinical presentation and demographic associations of thyrotoxicosis compared to hypothyroidism suggest unique entities with different etiologies. Thyroid irAEs, particularly overt thyrotoxicosis, are associated with increased immune toxicity in other organ systems, but also with longer progression-free and overall survival. Polygenic risk scores using susceptibility loci associated with autoimmune thyroiditis predict development of checkpoint inhibitor associated irAEs, suggesting potentially shared mechanisms underpinning their development. Our review will provide an up-to-date summary of knowledge in the field of thyroid irAEs. Major focus will be directed toward pathogenesis (including genetic factors shared with autoimmune thyroid disease), demographic associations, clinical presentation and course, treatment, and the relationship with cancer outcomes.

## Introduction

Immune checkpoint inhibitors (ICIs) are a rapidly expanding class of medications. At present, over 2000 clinical trials have been completed or are in progress to evaluate more than 30 different ICIs (1). The main immune checkpoint targets are cytotoxic T-lymphocyte antigen-4 (CTLA-4) and programmed cell death protein-1 (PD-1) or its ligand programmed cell death ligand-1 (PD-L1). The introduction of ICIs into cancer treatment algorithms has been transformative and durable responses are now possible in patients with previously limited therapeutic options. However, immune activation following ICI-treatment is associated with off target effects known as immune related adverse events (irAEs) (2). Any organ system may be affected and thyroid dysfunction is one of the most common ICI-related toxicities (3).

## Immune Checkpoint Physiology

Immune checkpoints are small molecules present on the cell surface of T-lymphocytes (4). Normally, T-cell activation induces immune checkpoint expression to limit the amplitude and duration of immune responses (4). Immune checkpoints are pivotal to regulate the immune response and prevent inappropriate immune activation, such as occurs in autoimmune disease (4). However, in some situations, immune checkpoints can be counterproductive, such as malignant tumors which activate immune checkpoints to evade immune mediated tumor lysis (3). Monoclonal antibodies antagonizing CTLA-4, PD-1, and other immune checkpoints have been developed to counteract such tumor evasion strategies and stimulate anti-tumor immune responses (4). These agents convey significant therapeutic benefit but are associated with wide ranging irAEs (2–4).

## Immune Related Adverse Events

The etiology of irAEs is yet to be fully elucidated. Potential mechanisms include activation of polyclonal T-cell populations, loss of regulatory T-cell function, and expansion/activation of self-reactive, antigen-specific T-cells (5). Irrespective of mechanism, ICI-associated irAEs are common, affecting up to 76% of treated patients (3). Any organ system can be affected, although there are distinct pharmacological class-specific differences between ICIs targeting CTLA-4 and those targeting PD-1 (3). Endocrine organs are commonly affected, with thyroid dysfunction, hypophysitis, insulin deficient diabetes, primary adrenal insufficiency, and hypoparathyroidism all reported following ICI-treatment (2, 6). In this review we will highlight the key features of thyroid irAEs and how our understanding of thyroid irAEs has informed our understanding of autoimmune thyroid disease more generally.

## Thyroid Immune Related Adverse Events

### Epidemiology

Thyroid irAEs are the most common endocrine toxicity related to ICI-treatment (6). From clinical trial data, over 10% of patients treated with ICIs develop a thyroid irAE (7). Higher rates are observed following treatment with PD-1 inhibitors relative to CTLA-4 inhibitors, with the highest rates following combined anti-PD-1 and anti-CTLA-4 treatment (7). Observational studies typically report higher rates of thyroid irAEs, usually due to inclusion of subclinical thyroid dysfunction that may be overlooked in clinical trials adverse events reporting (8–11). In the two largest observational studies of thyroid irAEs to date, 42-53% experienced an ICI-associated thyroid irAE (**Table 1**) (11, 12). Thyroid dysfunction was most common following combined anti-PD-1 and anti-CTLA-4 treatment (56%) and less frequent following anti-PD-1 (38%) and anti-CTLA-4 (25%) monotherapy (11).

**Table 1:** Summary of findings from the two largest studies of thyroid irAEs.

Study	Muir et al. (11)	Von Itzstein et al. (12)
**Number of patients**	1246 All patients had normal TSH prior to ICI-treatment and were free from pre-existing thyroid disease	1781 Many included patients had pre-existing thyroid disease prior to ICI-treatment (381 patients had abnormal TSH at baseline and 166 were receiving thyroxine; an additional 202 patients with normal TSH at baseline were also receiving thyroxine prior to ICI-treatment)
**ICI-therapy**; n (%) CTLA-4 PD-1/PD-L1 CTLA-4 + PD-1	165 (13) 705 (57) 376 (30)	56 (3) 1445 (81) 280 (16)
**Cancer type**; n (%) Melanoma Lung Kidney Other	1246 (100) - - -	238 (13) 512 (29) 338 (19) 599 (34)
**Patients developing a thyroid irAE**; n (%)	518 (42)	863 (53)
**Thyroid irAE subtype**; n (%)	Subclinical thyrotoxicosis n=234 (19); overt thyrotoxicosis n=154 (12); subclinical hypothyroidism without preceding thyrotoxicosis n=61 (5); overt hypothyroidism without preceding thyrotoxicosis n=39 (3)	TSH became elevated post ICI-treatment n=492 (30); TSH became low post ICI-treatment n=204 (13); TSH became both elevated and low post ICI-treatment n=167 (10)
**Patients progressing to hypothyroidism following an initial thyrotoxic phase**; n (%)	20/234 (9%) of patients with subclinical thyrotoxicosis; 91/154 (59%) of patients with overt thyrotoxicosis	Not reported
**Patients developing permanent hypothyroidism requiring initiation of thyroid hormone replacement**; n (%)	n=7 (3) patients with subclinical thyrotoxicosis; n=66 (43) patients with overt thyrotoxicosis; n=0 (0) patients with subclinical hypothyroidism; n=29 (74) patients with overt hypothyroidism	n=267 (15) new patients required thyroxine in addition to thyroxin continuation for the n=368 (21) patients already receiving thyroxine at baseline
**Thyroid irAE kinetics**; median (IQR)	***Time to onset:*** subclinical thyrotoxicosis – 8 wks (4-14); overt thyrotoxicosis – 5 wks (2-8); subclinical hypothyroidism – 10 wks (3-27); overt hypothyroidism – 14 wks (8-25) ***Time to restoration of euthyroidism:*** subclinical thyrotoxicosis – 4 wks (1-8); overt thyrotoxicosis – 12 wks (7-24); subclinical hypothyroidism – 3 wks (1-8); overt hypothyroidism – 10 wks (1-24)	Not reported
**Patients with positive anti-TPO and anti-Tg antibodies**	TPOAb was positive at baseline in 27/163 (17%) patients; 27/27 patients with TPOAb developed a thyroid irAE TgAb was positive at baseline in 42/163 (26%) patients; 41/42 patients with TgAb developed a thyroid irAE 22 of the above patients were positive for both TPOAb and TgAb	Not reported
**Patient and disease characteristics associated with development of thyroid irAEs**	Female sex, younger age, absence of brain metastases, combined CTLA-4 + PD-1 ICI-treatment ***Overt thyrotoxicosis*** *was uniquely associated with the development of extra-thyroidal, multi-system, and severe irAEs which were not associated with other thyroid irAE subtypes*	Female sex, Caucasian ethnicity, primary kidney malignancy*, combined CTLA-4 + PD-1 ICI-treatment
**Effect of thyroid irAE on cancer survival**	Presence of any thyroid irAE (all subtypes) was not associated with a benefit in OS or PFS However, the thyroid irAE subtype of ***overt thyrotoxicosis*** was associated with significant improvement in OS (HR 0.57, 95% CI 0.39-0.84) and PFS (HR 0.68, 95% CI 0.49-0.94) Improvements in OS and PFS were not present with other subtypes of thyroid irAE when tested individually	Survival was improved in patients with a thyroid irAE relative to those without (median OS 43 mths vs 26 mths); The highest OS was in patients with a normal TSH at baseline and an abnormal TSH after ICI-treatment (41 mths); The lowest OS was in patients with an abnormal TSH at baseline and a normal TSH during ICI-treatment (12 mths) In multivariate analyses, abnormal TSH prior to ICI-treatment was associated with worse survival (HR 1.62, 95% CI 1.30-2.02); initiation of thyroxine after ICI-treatment was associated with improved survival (HR 0.62, 95% CI 0.44-0.88)

irAE, immune related adverse event; TSH, thyroid stimulating hormone; ICI, immune checkpoint inhibitor; CTLA-4, cytotoxic T-lymphocyte antigen-4; PD-1, programmed cell death protein-1; PD-L1, programmed cell death ligand-1; IQR, interquartile range; TPOAb, thyroid peroxidase antibody; TgAb, thyroglobulin antibody; OS, overall survival; PFS, progression free survival.

### Clinical Manifestations

Thyroid irAEs are typically identified incidentally during routine monitoring of thyroid function as part of ICI-treatment protocols (7). Most thyroid irAEs present as a painless thyroiditis with transient thyrotoxicosis (6, 7, 11, 13). In patients with more severe (ie. biochemically overt) thyrotoxicosis, a hypothyroid phase often follows the initial thyrotoxicosis and over 40% of these patients will develop permanent hypothyroidism requiring thyroid hormone replacement (11). A smaller number of patients can present with primary hypothyroidism without a preceding thyrotoxic phase.

Onset of thyrotoxicosis usually occurs within 3 months of first ICI-exposure (6, 7). Overt and subclinical thyrotoxicosis have phenotypic differences in their presentation and disease associations. Onset of overt thyrotoxicosis typically occurs earlier and persists longer than subclinical cases (11). Overt thyrotoxicosis has also been associated with higher baseline levels of anti-thyroid antibodies which increase during ICI-inhibitor treatment (14). Treatment related changes in antibody titer have not been observed in other subtypes of thyroid irAE (14). Patients who develop overt thyrotoxicosis are more likely to develop severe irAEs and to experience two or more irAEs in extra-thyroidal organ systems (11).

Isolated hypothyroidism without a preceding thyrotoxic phase can occur. Most cases of isolated hypothyroidism are biochemically subclinical and may be related to non-ICI factors such as non-thyroidal illness syndrome. Subclinical hypothyroidism without a preceding thyrotoxic phase typically normalizes spontaneously without need for thyroid hormone replacement (11, 15). Overt hypothyroidism can have a late-onset months to years after starting ICI-treatment (7). In contrast with subclinical hypothyroidism, overt hypothyroidism is usually permanent and lifelong treatment with thyroxine is required in most cases (6, 7, 11).

### Risk Factors and Disease Associations

Identification of reliable thyroid irAE risk factors has been elusive. To date, no reliable risk factors of thyroid irAEs have been identified, although clinical and biochemical associations have been documented in retrospective cohort studies (7). Female sex was associated with thyroid irAEs in the two largest studies to date (**Table 1**), although to a lesser degree than the roughly 8:1 female preponderance observed in autoimmune thyroid disease developing outside of the ICI-treatment setting (11, 12). Prevalence of baseline TPOAb and TgAb positivity is higher in patients who develop a thyroid irAE compared to those who do not (11, 16). Anti-thyroid antibody positivity is particularly associated with overt thyroid dysfunction (11, 16) and titers of TPOAb and TgAb can increase significantly during ICI-treatment in patients that develop overt thyrotoxicosis, which is not observed in patients with other subtypes of thyroid irAE (14). A complete list of demographic and biochemical factors that have been associated with development of thyroid irAEs are summarized in **Table 2**.

**Table 2:** Demographic and biochemical factors associated with development of thyroid irAEs.

Female sex (11, 12) Younger age (11) Caucasian ethnicity (12) Combined CTLA-4 + PD-1 treatment (11, 12) Longer duration of anti-PD-1 treatment (16) Higher body mass index (17) Renal cell carcinoma* (12) Baseline TSH (8, 16–18) Baseline TPOAb positivity; treatment related increase in TPOAb titer (14, 16, 19) Baseline TgAb positivity; treatment related increase in TPOAb titer (14, 16, 18, 19)

CTLA-4, cytotoxic T-lymphocyte antigen-4; PD-1, programmed cell death protein-1; TSH, thyroid stimulating hormone; TPOAb, thyroid peroxidase antibody; TgAb, thyroglobulin antibody; *Likely confounded by concomitant use of tyrosine kinase inhibitors in combination with ICIs.

### Pathophysiology

The etiology and biological drivers of irAEs remain elusive. It is possible that patients who experience irAEs may share a premorbid immunological state prior to ICI-initiation. A recent study using mass cytometry time of flight analysis identified higher levels of activated CD4+ memory T-cells and increased diversity in the T-cell receptor as pretreatment predictors of severe irAEs irrespective of the organ involved (20). These same factors also occurred with higher frequency in non-ICI treated patients with autoimmune disease relative to healthy controls, suggesting that irAEs may result from unmasking a latent or subclinical autoimmune process which predates ICI-initiation (20).

Traditionally, thyroid irAEs were thought to be a single entity spanning a spectrum of disease including isolated mild (asymptomatic or minimally symptomatic) thyrotoxicosis to biphasic thyroiditis to isolated overt and severe hypothyroidism. This indeed may be the case, although more likely thyroid irAEs arise *via* multiple mechanisms as evidenced by phenotypic differences in presentation, antithyroid antibody positivity rates, and differential association with cancer survival outcomes (11).

Genetic factors associated with lifetime risk of autoimmune thyroid disease have been implicated in the risk of developing thyroid irAEs during ICI-treatment (21, 22). Key loci have been implicated from genes regulating the immune response such as CD69 (T-cell activation), LPP (B-cell maturation), CTLA-4 (T-cell priming) and PTPN22 (T-cell and B-cell receptor signaling). When these genes and others are combined into a polygenic risk score they can identify subgroups of patients at >6-fold increased risk of thyroid irAEs and identify patients at a lower risk of cancer death (21).

The PD-1/PD-L1 axis is significantly implicated in autoimmune thyroid disease (23). Patients with autoimmune thyroiditis and Graves’ disease experience a moderate increase in circulating PD-1 positive T-cells and marked increase in intrathyroidal PD-1 positive T-cells (23). Thyroid follicular cells also show high levels of PD-L1 expression, whereas multinodular goiter (non-autoimmune thyroid disease) has negligible basal expression of PD-L1 (23). The presence of high PD-L1 expression in autoimmune thyroid disease may explain why most cases are slowly progressive, as the immune stimulating effects of intrathyroidal PD-1 positive T-cells are kept in check. PD-1 positive lymphocytes and PD-L1 expression are similarly increased following ICI-treatment. However, different to autoimmune thyroid disease, blockade of the PD-1/PD-L1 axis blunts the protection against progressive immune mediated destruction *via* and therefore thyroid dysfunction is more rapidly progressive following ICI-treatment than in other autoimmune thyroid conditions (23, 24).

### Management

#### Screening

Thyroid function should be measured at baseline and repeated at 6-weekly intervals following commencement of ICI-treatment in asymptomatic patients. Additional measures of TSH and FT4 can be undertaken for case detection in patients that develop signs or symptoms of thyroid dysfunction outside this window (25). Most cases of clinically significant thyroid dysfunction will occur within 6-months of ICI-commencement (7). Therefore, monitoring frequency can be relaxed after the 6-month mark although continued periodic monitoring (3-6 monthly) to detect late onset cases is recommended.

#### Diagnosis

As most cases of thyroid irAE are asymptomatic, diagnosis is most commonly *via* thyroid function monitoring in asymptomatic patients (7). Thyrotoxicosis is diagnosed in the setting of a low TSH with a normal or elevated FT4. Thyrotoxicosis often occurs as the initial phase of a biphasic thyroiditis and is followed by progression to hypothyroidism (elevated TSH, low FT4). More rarely, isolated hypothyroidism without a preceding thyrotoxic phase can occur. Unlike thyrotoxicosis, isolated hypothyroidism may present months to years after initiation of ICI-treatment. Special attention should be paid to the finding of an inappropriately low TSH in combination with a low or low-normal FT4. This pattern of results should alert the clinician to the possibility of hypophysitis, particularly in the setting of a CTLA-4 inhibitor. When hypophysitis (central hypothyroidism) is suspected, the remaining pituitary hormone axes should be interrogated urgently to exclude cortisol and other hormone deficiencies prior to initiation of thyroid hormone replacement.

#### Treatment

**Figure 1:**
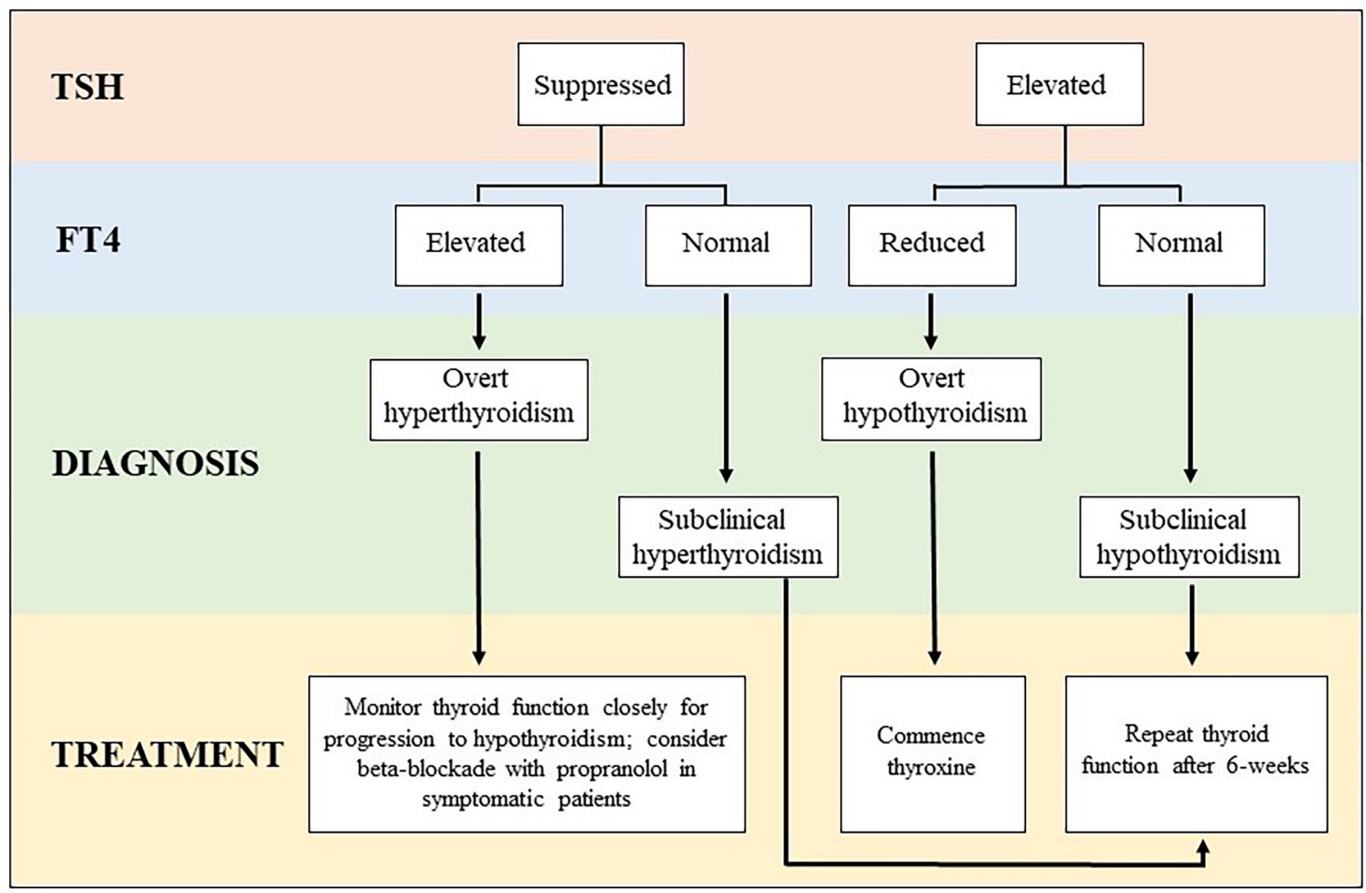
Algorithm for classification and management of thyroid irAE subtypes. *When overt thyrotoxicosis is prolonged or severe, additional investigation with TSH receptor antibody (TRAB) and a thyroid uptake scan should be considered to exclude other etiologies of thyrotoxicosis (ie. Graves’ disease, toxic adenoma, etc).

For asymptomatic or minimally symptomatic thyrotoxicosis, treatment is usually not required. Temporary use of beta blockers such as propranolol may be considered in symptomatic patients. ICI-treatment can be continued, and most cases will resolve spontaneously within days to weeks. Thyrotoxicosis lasting longer than 6-weeks or with additional features (goiter, thyroid bruit, exophthalmos) should have TSH-receptor antibody level (TRAB) measured to exclude Graves’ disease (25). In severe or atypical presentations, imaging with a thyroid uptake scan can also be performed to differentiate between ICI-mediated thyroiditis and other etiologies of thyrotoxicosis. Thyrotoxicosis with marked symptoms or life-threatening complications is rare and necessitates suspension of ICI-treatment to allow for prompt investigation and specialist led treatment (25). Following identification of thyrotoxicosis, TSH and FT4 should be monitored every 2-3 weeks until recovery of normal thyroid function, as many patients will experience a biphasic illness which progresses to hypothyroidism. Regular monitoring allows early detection of hypothyroidism, which does not recover in the majority of cases (11). In the setting of overt hypothyroidism, treatment with thyroxine can be initiated, particularly when the TSH is >10 mIU/L, as recovery is highly unlikely in this setting (11). Alternatively, TSH and FT4 can be repeated after 4-6 weeks and if hypothyroidism persists, treatment can be initiated at that point (25). Thyroxine doses are typically higher for patients with ICI-mediated hypothyroidism than for Hashimoto’s thyroiditis, suggesting that gland destruction is complete in most cases of ICI-mediated thyroiditis whereas it is slowly progressive with some residual production of endogenous thyroid hormone in patients with Hashimoto’s thyroiditis (26). A simplified algorithm for the classification and management of thyroid irAEs is presented in **Figure 1**.

### Special Situations

#### Pre-existing Hypothyroidism

Patients with established hypothyroidism may experience transient effects on thyroid function following ICI-treatment (7). Compensated hypothyroidism can be exacerbated requiring initiation or increased doses of thyroxine. More rarely, patients with hypothyroidism may paradoxically experience a transient period of thyrotoxicosis requiring temporary suspension of thyroid hormone (27).

#### Graves’ Disease and Thyroid Eye Disease

Graves’ disease (GD) has been reported following ICI-treatment (6). Whether it occurs *de novo* related to ICI-treatment, is unmasked by ICI-treatment, or is a coincidental occurrence remains unknown. Similarly, thyroid eye disease (TED) has been reported (28). Reactivation or progression of GD and TED is possible following initiation of ICI-treatment and in patients with known GD or TED, close consultation with an experienced endocrinologist and ophthalmologist is important for early identification and treatment.

### Prognostic Implications

Observational studies repeatedly document an association between irAEs and improvement in cancer outcomes (29, 30). Owing to the frequency of thyroid irAEs, they are among the most well studied and strongly associated with improvement in progression free survival (PFS) and overall survival (OS) (31–33). Reported benefits are large with robust hazard ratios that are maintained following correction for immortal time bias (31, 34). However, emerging data suggest thyroid irAE subtypes are not equivalent in their association with cancer outcomes. When thyroid irAEs are classified by severity, biochemically overt thyroid dysfunction is more strongly associated with improved PFS and OS than subclinical thyroid dysfunction (11, 35, 36). Our previous work suggests this may be limited to overt thyrotoxicosis, as no survival benefit was observed in patients with overt hypothyroidism without a preceding thyrotoxic phase (11). Permanent thyroid dysfunction requiring initiation of thyroid hormone replacement has also been associated with improved cancer outcomes (12, 37). However, an ultimate requirement for thyroxine may have been a surrogate marker for a preceding phase of overt thyrotoxicosis rather than a beneficial prognostic factor in of itself. The significance of antithyroid antibodies on cancer outcomes following ICI-treatment is unknown and current data has conflicting results (35, 38). Given the high prevalence of anti-thyroid antibodies in patients who develop overt thyrotoxicosis, an association is likely and prospective study into potential utility as a predictive biomarker is warranted (14).

Interestingly, abnormal TSH level (elevated or low) prior to initiation of ICI-treatment has been associated with inferior cancer outcomes irrespective of thyroid function changes post-ICI initiation in one study (12). However, these results may have been confounded as significantly more patients with abnormal baseline TSH were receiving tyrosine kinase inhibitor treatment for metastatic renal cell cancer, which is known to significantly affect thyroid function (12). Abnormal TSH prior to ICI-treatment may also be a function of sicker patients displaying the non-thyroidal illness syndrome. Therefore, at present it is not known whether baseline thyroid function is truly associated with worse outcomes or if it simply reflects patients with a more aggressive cancer or a physiologic response to chronic illness in a less well patient at commencement of ICI-treatment.

## Future Directions

Further research is required to better inform our understanding of thyroid irAEs and their relationship to thyroid autoimmune conditions occurring outside the cancer immunotherapy setting. Enhanced characterization of the immune phenotype and T-cell subsets driving thyroid irAEs may allow for identification of patients at risk of thyroid (and other) irAEs and inform future research to uncouple the efficacy of ICIs from the risk of developing treatment related irAEs. Understanding the effects of immune checkpoint inhibition on the thyroid gland could also inform use of these agents for treatment of primary thyroid malignancies and prevention of autoimmune thyroid disease.

## Conclusions

Thyroid irAEs are a frequent complication of anti-PD-1 based ICI-treatment. Recent work has begun to unravel the molecular mechanisms underpinning development and their relationship to other autoimmune thyroid conditions. Regular monitoring of thyroid function during ICI-treatment is required, as although most cases of thyroid irAEs are asymptomatic and manageable, severe presentations with thyroid storm or myxedema can occur. Development of thyroid irAEs is associated with improved PFS and OS, especially overt thyrotoxicosis which may be a surrogate marker of patients experiencing a more robust immune response to ICI-treatment. When hypothyroidism occurs, it is usually permanent and treatment with thyroid hormone replacement will be required. Familiarity with thyroid irAEs among health professionals is important to facilitate efficient diagnosis and appropriate treatment of this increasingly common thyroid disease.

## Author Contributions

All authors listed have made a substantial, direct, and intellectual contribution to the work. All authors approved it for publication.

## Conflict of Interest

The authors declare that the research was conducted in the absence of any commercial or financial relationships that could be construed as a potential conflict of interest.

## Publisher’s Note

All claims expressed in this article are solely those of the authors and do not necessarily represent those of their affiliated organizations, or those of the publisher, the editors and the reviewers. Any product that may be evaluated in this article, or claim that may be made by its manufacturer, is not guaranteed or endorsed by the publisher.
